# Open-Access Web-Based Gamification in Pharmacology Education for Medical Students: Quasi-Experimental Study

**DOI:** 10.2196/73666

**Published:** 2025-12-05

**Authors:** Lujain Aloum, Halah Ibrahim, Senthil Kumar Rajasekaran, Eman Alefishat

**Affiliations:** 1 Department of Medical Sciences, College of Medicine and Health Sciences, Khalifa University of Science and Technology, Abu Dhabi, United Arab Emirates; 2 Department of Biopharmaceutics and Clinical Pharmacy, Faculty of Pharmacy, University of Jordan, Amman, Jordan; 3 Department of Biomedical and Translational Sciences, Carle Illinois College of Medicine, University of Illinois Urbana-Champaign, 506 South Mathews Avenue, Urbana, IL, 61801, United States, 1 2172495545

**Keywords:** pharmacology, gamification, open access, medical education, medical students

## Abstract

**Background:**

Medical education continues to favor didactic lectures as the predominant method of instruction. However, in recent years, there has been a shift toward active learning methodologies such as gamification.

**Objective:**

This study aimed to describe the implementation of 3 open-access, web-based pharmacology games tailored for medical students: *Cross DRUGs*, *Find the DRUG*, and *DRUGs Escape Room*. The study also evaluated the impact of gamification on knowledge retention, student engagement, and learning experience in pharmacology education.

**Methods:**

We used a quasi-experimental design to examine the effects of gamification on knowledge retention by comparing pretest and posttest scores between the gamer and control groups. Each week, students self-selected into either the gamer group or the control group based on personal preference. All students were provided with online access to the same lecture slides. Students in the control group completed both the pretest and posttest but did not play any of the games. A survey was administered to assess students’ perceptions of gamification as a learning tool.

**Results:**

Of the 72 students enrolled in the course, 49 (68%) agreed to participate, with 40 (56%) students completing both the pretest and posttest and being included in our analysis. As participation could vary weekly, an individual student might have appeared in both groups across different weeks, resulting in 59 gamer sessions and 20 control sessions. The mean pretest scores were 6.05 (SD 2.31) for the control group and 6.20 (SD 2.13) for the gamer group. The mean posttest scores were 6.90 (SD 2.02) for the control group and 8.47 (SD 1.30) for the gamer group. The gamer group exhibited significantly improved posttest scores (*P*=.006), while the control group did not (*P*=.21). Most respondents (25/30, 83%) found the games enjoyable and agreed that the games effectively helped them understand pharmacological concepts (24/30, 80%). Additionally, 70% (21/30) of students believed they learned better from the gaming format than from didactic lectures. Most favored a blended approach that combines lectures with games or case studies.

**Conclusions:**

Gamification can serve as an effective complementary teaching tool for helping medical students learn pharmacological concepts.

## Introduction

Medical education continues to favor didactic lectures as the predominant method of instruction [1,2], even though this traditional approach fosters passive learning, reinforces teacher-centeredness [3], and yields lower rates of knowledge retention compared to other approaches [4]. In recent years, there has been a growing shift toward innovative and active learning methodologies, among which gamification has emerged as a promising strategy [5].

In its simplest terms, gamification involves integrating game elements into nongame contexts [6,7]. Gamification in education is grounded in several learning theories, including humanistic learning and adult learning theories [8,9]. It incorporates features such as goal setting, incremental challenges, immediate feedback, progression systems, and rewards to foster deeper involvement in learning tasks [10,11]. Gamification has been shown to enhance motivation, improve academic achievement, and foster social interaction, thereby supporting its use as an effective teaching tool [11-13].

For Generation Z learners, considered to be “digital natives,” gamification may be the most appropriate pedagogical method as it meets Generation Z’s familiarity and immersion in digital platforms [14-17]. Furthermore, their extensive exposure to technology may have resulted in the development of constrained attention spans, a preference for visual and kinesthetic learning activities, and a need for immediate feedback [14]. This trend in learning preferences requires educators to transition from content providers to facilitators of learning who can skillfully leverage technology, including gamification, to boost student engagement and motivation [14,18,19].

Although many studies have demonstrated that gamification enhances the learning experience and increases knowledge retention [20-24], they have been criticized for publication bias and for consistently reporting positive outcomes in health professions education programs [25]. Methodological concerns have also been raised, with most studies lacking proper control groups, raising doubts about the reliability of the evidence supporting the impact of gamification on learning outcomes. A systematic review on gamification underscored the need for more rigorous research designs with defined control groups to accurately assess the benefits of gamification in health professions education [25]. This study aimed to describe the implementation of 3 open-access, web-based pharmacology games for medical students and to explore the impact of these games on knowledge retention and student experience.

## Methods

### Setting and Study Design

Khalifa University College of Medicine and Health Sciences is a 4-year postgraduate entry medical school in the United Arab Emirates. Many of the students do not follow the traditional premedical route and instead have engineering backgrounds. The pharmacology course is a mandatory 4-week course for all first-year medical students. It typically requires memorization of large amounts of information, which presents an academic challenge for students. Recognizing that a lecture-based delivery of the pharmacology course might not benefit students who may struggle with rote memorization of a long list of facts, we sought innovative teaching methods to enhance the learning experience.

We conducted a quasi-experimental study to examine the effects of gamification on student experience and knowledge retention during the first 3 weeks of a 4-week pharmacology course; the final exam week was excluded. Quasi-experimental designs are commonly used in medical education when randomization is not feasible due to the practical and ethical constraints of real-world educational settings [26]. We used a pretest-posttest, nonrandomized control group design. This approach is consistent with the framework of nonequivalent group designs where participants are not randomly assigned but are compared based on pretest and postintervention outcomes to infer the impact of the intervention [27,28].

### Ethical Considerations

The CONSORT-EHEALTH (Consolidated Standards of Reporting Trials of Electronic and Mobile Health Applications and Online Telehealth) checklist was used to guide our reporting [29]. The study was approved by the Institutional Review Board of Khalifa University of Science and Technology (H20-036). At the start of the course, each student received an email invitation to participate in the study along with an informed consent form, and all participants provided informed consent. The email described the study’s purpose and explained that it was anonymous and confidential. Participation was voluntary, and no incentives were offered. Students were informed that they could withdraw from the study at any time without any consequences. Student participation and data deidentification were handled by a study coordinator who was not involved in student teaching or grading.

### Participants and Group Allocation

Students were allowed to self-select into either the gamified (gamer) or nongamer (control) group each week based on their personal preference. While this self-selection limited random assignment, it is a pragmatic approach for studying educational interventions that mirror real-world classroom decision-making [30]. Given the weekly self-selection process, a single student could participate in both types of sessions across different weeks, resulting in 59 gamer sessions and 20 control sessions, reflecting learning sessions rather than unique participants.

### Game Design

We chose 3 open-access, web-based game formats—crossword puzzle, word search, and escape room—because they align with adult learning theories by incorporating clear goals, incremental challenges, and immediate feedback [7]; require minimal technological infrastructure; and support asynchronous, self-directed learning suited to Generation Z’s digital preferences. *Cross DRUGs* (Multimedia Appendix 1), based on a crossword game, was generated via an online crossword puzzle generator available on the Education website [31]. *Find the DRUG* (Multimedia Appendix 1) was based on the Hunting Words game and was produced using an online word search puzzle generator available on the Educolorir website [32]. *DRUGs Escape room* (Multimedia Appendix 1), created via Google Forms using the EduGame template, required students to solve a series of sequential, drug-related clues to progress through the game.

Each game tested students on the drugs covered during the preceding week of the pharmacology course, including the mechanisms of action, primary indications, and clinically relevant side effects. Two authors, who were faculty members in the pharmacology course, independently drafted each game’s questions based on weekly learning outcomes. A third author, also a course instructor, reviewed the games for accuracy and curricular alignment. Games were single-player and time-limited, but students were allowed unlimited attempts. Students were given 48 hours to participate in the game, and only 1 attempt was required for inclusion. Upon completion, correct answers and brief explanations were provided via the learning management system.

### Assessment

All students received the same curriculum. At the end of each week and before game access, all students completed a 9-item multiple-choice pretest. The pretest was conducted to assess the pharmacological knowledge of students regarding the content covered that week and to ensure the homogeneity of the knowledge acquired by both the control and gamer groups. Instructions on how to access the games were provided via email, along with a reminder encouraging students to play the game. At the end of the 48-hour period for game completion, a matched 9-item posttest was released to both groups. Students in both the gamer and control groups accessed the posttest at the same time. The pretest and posttest contained identical items to leverage spaced repetition principles and measure knowledge gains attributable to gameplay. Spaced repetition involves revisiting material at intervals to reinforce understanding over time [33]. The tests were formative and did not influence course grades. Students in the control group had online access to the lecture slides that were also available to the gamer group, while the latter engaged in gameplay.

### Survey Instrument

At the end of the course, all students who participated as gamers were asked to complete an online questionnaire assessing enjoyment, perceived learning benefits, and preferences for gamification versus lectures using a 5-point Likert scale (1=strongly agree to 5=strongly disagree). The survey also evaluated the importance of incorporating a reward system into gamification and compared gamification to other teaching methodologies. The postgame survey was administered only to students in the gamer group, as it focused on perceptions of the gamified activities. Because the control group did not receive any additional instructional method beyond lecture slides, the questionnaire was not applicable. We calculated Cronbach α for internal consistency (overall α=0.88; reward-related items α=0.65) [34]. Deleting any question caused the α coefficient to decrease, suggesting that each question contributed to the overall reliability, as shown in Multimedia Appendix 2.

### Data Analysis

We performed analyses in R (version 4.2.3; R Foundation for Statistical Computing). In line with prior studies on the impact of games on learning outcomes, score differences (posttest minus pretest) between control and gamers were used to assess the effectiveness of the games [35]. The Shapiro-Wilk test and Mann-Whitney test were performed using the built-in stats package, and Cronbach α was calculated using the psych package. To assess the normality of the pretest and posttest scores, the Shapiro-Wilk test and histogram inspection were conducted. Because the scores were not normally distributed, we compared groups with the Mann-Whitney test (Wilcoxon rank-sum test with continuity correction) and set statistical significance at *P*<.05.

## Results

**Figure 1.**
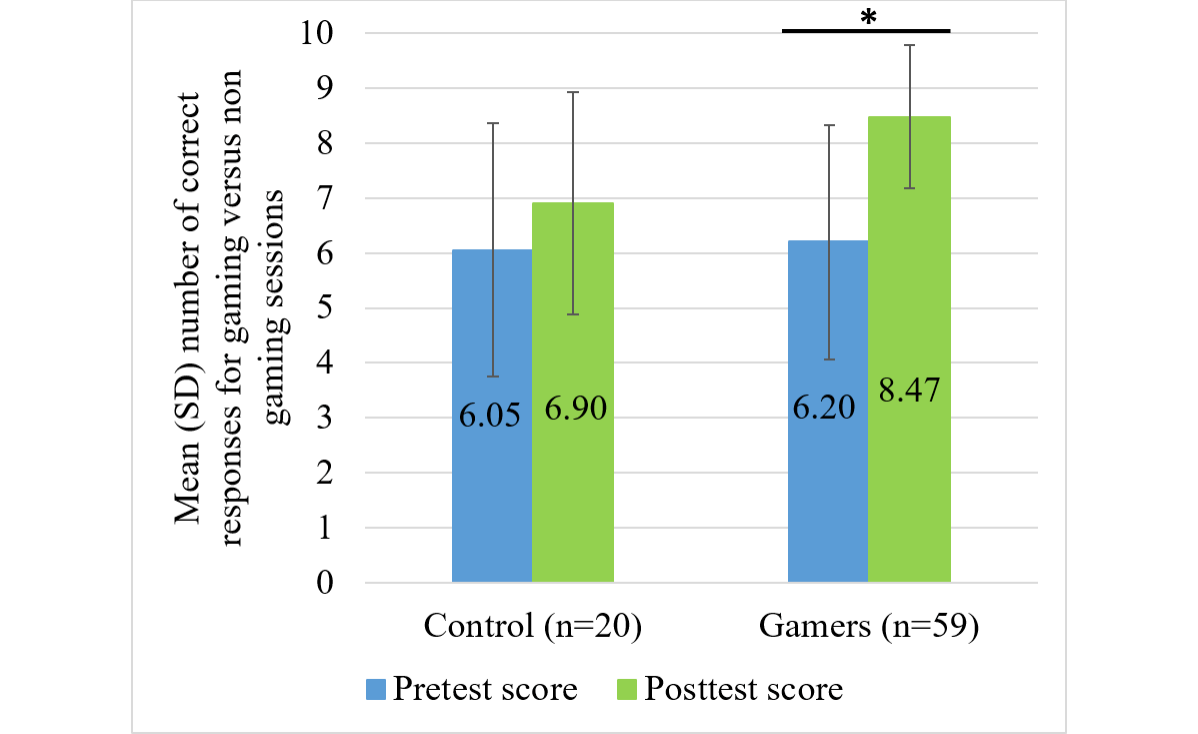
Mean (SD) number of correct responses on the pretest and posttest for students who did not play the game (control group; n=20 sessions) and those who played the game (the gamer group; n=59 sessions). Each data point represents a learning session rather than a unique participant. The highest attainable score was 9. *P=.006.

Of the 72 students enrolled in the course, 49 (68%) agreed to participate, with 40 (56%) students completing both the pretest and posttest and being included in our analysis (Figure 1). As students self-selected into the gamer or control group each week, there were 59 gamer sessions and 20 control sessions. The average number of attempts per gamer was 3.4 (SD 0.85). Participant demographics are presented in Multimedia Appendix 3. The self-identified mean age was 23.9 (SD 2.69) years for the control group and 24.0 (SD 1.79) years for the gamer group. Students had educational backgrounds in engineering or scientific disciplines (*others*), typically holding bachelor’s degrees in chemistry, biology, biochemistry, or psychology. None of the students reported prior educational experience in pharmacy or pharmacology.

Figure 1 displays the average pretest and posttest scores for both groups. The mean pretest scores were 6.05 (SD 2.31) for the control group and 6.20 (SD 2.13) for the gamer group. The mean posttest scores were 6.90 (SD 2.02) for the control group and 8.47 (SD 1.30) for the gamer group. The gamer group demonstrated a significant improvement in posttest scores (*P=*.006), whereas the control group did not show a statistically significant change (*P=*.21).

**Figure 2.**
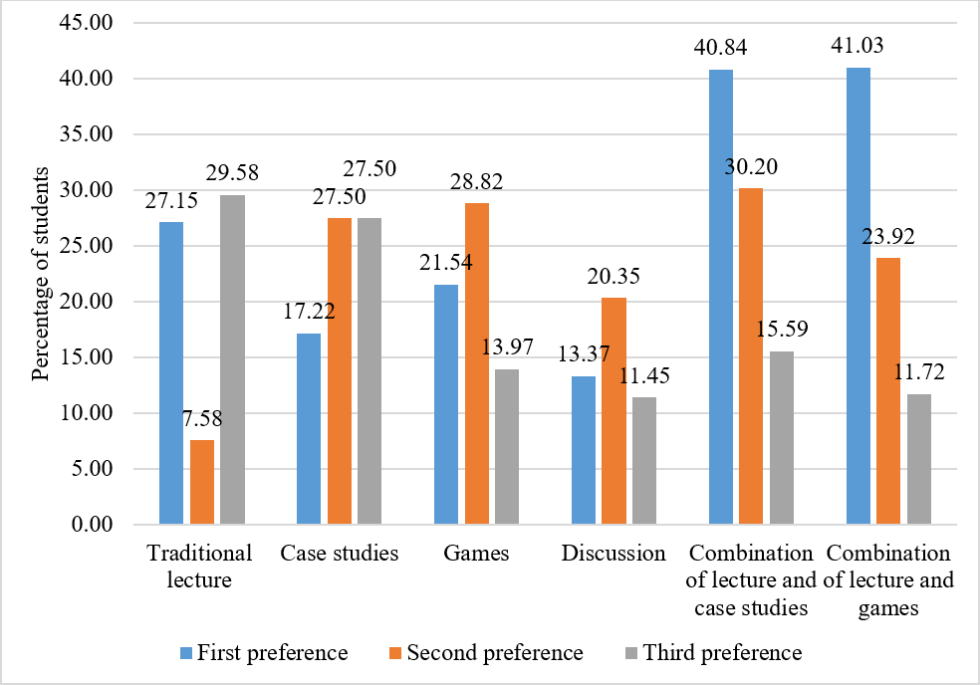
Students’ preferences for different instructional formats in pharmacology education, presented as percentages for first, second, and third choices (n=30).

Survey results demonstrated positive perceptions of gamification as a learning tool. Of the 30 students surveyed, 24 (80%) agreed that the games were an efficient way to understand pharmacological concepts, and 25 (83%) reported that the games were enjoyable. Most students (21/30, 70%) believed that they learned better from the gaming format than from didactic lectures, and 67% (20/30) agreed that awarding points, such as extra credits, for game-related activities would be beneficial. Less than half of the respondents (12/30, 40%) agreed with allocating prizes to game winners, while the remaining students were evenly divided between neutral and disagreement (Table 1). Surveyed students favored a teaching methodology that combines lectures with games or case studies, with the highest percentage of students (9/30, 30%) preferring lectures as their third preference (Figure 2).

**Table 1.** Students’ responses to 5 evaluation statements on gamified pharmacology learning activities (n=30). The statements assessed perceived effectiveness, enjoyment, learning preferences, and attitudes toward rewards.

Statements	Strongly agree or agree, n (%)	Neutral, n (%)	Strongly disagree or disagree, n (%)
The game was an effective way to learn pharmacological concepts	24 (80)	3 (10)	3 (10)
I enjoyed the game	25 (83)	3 (10)	2 (7)
I learn better in a game format than in a didactic lecture	21 (70)	3 (10)	6 (20)
I feel that prizes should be awarded to the winners of the games	12 (40)	9 (30)	9 (30)
Points (either as extra credit or incorporated into the overall grading scheme) should be associated with game activities	20 (67)	4 (13)	6 (20)

## Discussion

We integrated 3 low-cost, easily implemented, open-access pharmacology games into a pharmacology course for medical students. Students who engaged in the game-based learning approach demonstrated better knowledge retention with a significant improvement in test scores compared to the control group. Most students also perceived the games as effective, enjoyable, and preferable compared to traditional lectures. Many students suggested that a blended instructional model combining didactic sessions with interactive games would optimize learning by balancing content delivery with engaging reinforcement.

Our decision to implement gamification in pharmacology education was partly driven by the academic profile of our students, many of whom came from engineering backgrounds. These students often prefer spontaneous, pragmatic, and concrete learning styles with hands-on, goal-oriented, and sequential tasks. Research has shown that engineering students tend to favor trial-and-error learning; practical application; and structured, step-by-step problem-solving approaches [36]. These characteristics made them well suited for gamified learning environments that emphasize active participation and immediate feedback. In line with this, we selected crossword puzzles, word search activities, and web-based escape rooms, as they require minimal setup and support self-directed, asynchronous learning [37,38]. By breaking up complex pharmacology concepts into brief game sessions with immediate feedback, students experienced incremental successes that built self-efficacy and intrinsic motivation [25,39]. Furthermore, the variety of game formats helped sustain attention and catered to diverse learning preferences within the student cohort.

Two recent systematic reviews in pharmacy education and higher education reported that the most common type of research methodology was pretest and posttest evaluation [35,40]. Among the 3 games we implemented, the crossword and word search games lack supporting evidence in the literature that uses pretest and posttest scores [24,38,41]. In contrast, escape room–based strategies have been more rigorously evaluated, frequently using pretest and posttest to quantify knowledge gains. Consistent with our findings, several studies reported substantial improvements in posttest scores among pharmacy and medical students following escape room activities, although many lacked control groups [20,42-44]. While different in design, other gamified learning tools have also demonstrated improved knowledge retention. For instance, second-year medical students who played “Who Wants to be a Physician”—a game inspired by the TV show “Who Wants to be a Millionaire”—achieved higher posttest scores compared to peers who attended traditional tutorial sessions [45]. Similarly, both preclinical and final-year medical students and residents who engaged in board or card games showed posttest score improvements [46-48]. Additionally, the “Pharmacotrophy” tournament, which incorporated Kahoot-based quizzes and in-person matches, significantly enhanced PharmD students’ knowledge acquisition, likely due to its incorporation of fun elements and a relaxed, competitive environment [49]. These findings suggest that gamified approaches can be effectively integrated across various stages of medical education, from preclinical training to residency.

A common methodological limitation across many gamification studies is the lack of a control group, which limits the ability to draw causal inferences [20,24,38,41-44,46-48,50]. Notably, a systematic review of pharmacy education found that just 2% of studies included a control arm [40]. Our study addressed this gap by incorporating a clearly defined control group each week, enabling us to objectively demonstrate that game-based learners achieved significantly greater knowledge gains than their nongamer peers.

Consistent with previous research, most students perceived the games as efficient, enjoyable, and of more educational value than conventional didactic lectures. Students in previous studies found gamified learning methods, such as the diabetes board game, virtual escape room, crossword puzzles, and “Who Wants to be a Physician,” to be enjoyable ways to learn pharmacology, often citing increased engagement and confidence in the subject matter [20,24,38,41,45,46]. Similarly, use of the Kahoot platform increased medical student motivation and participation [51]. In our study, more than 80% of gamers agreed that the puzzles and escape room enhanced their understanding of pharmacological concepts and enjoyment of the subject. These findings underscore that well-designed gamification can meaningfully enrich medical education by providing opportunities for interactive learning.

Although students enjoyed the games, many favored a blended approach that combines interactive activities with conventional lectures. This preference suggests that while gamification can foster engagement, learners may not yet be ready to replace lectures entirely, especially within a traditionally structured curriculum. Our voluntary, supplemental design likely contributed to the positive findings by allowing motivated students to opt in without penalizing others. One study found that the impact of gamification depended on participant personality traits, suggesting that it was not beneficial for all learners [52], with some students considering games inefficient or tedious [53]. The moderate-enjoyment hypothesis further cautions that excessive game elements can overload cognitive resources, reducing learning gains beyond an optimal point [54]. Accordingly, we aimed to balance play and learning—using play to complement and reinforce complex information to increase learning efficiency, rather than as a stand-alone replacement for didactic teaching. This strategy aligns with recent recommendations to incorporate game-based learning as an optional strategy and only for tedious or difficult concepts [53]. A recent review supported the use of game-based learning as complementary tools, as it noted that the long-term applicability of these methods requires further exploration [5].

It is notable that the 3 games used in this study deliberately centered on a single game element—“rules and goals”—to provide clear objectives for each activity without introducing confounding game elements such as competition, narratives, or collaboration [55]. By focusing solely on structured challenges with immediate feedback, we could more confidently attribute the observed gains in retention and engagement to goal-oriented game elements [25]. We intentionally omitted competition, a common gamification feature [13,56-58], because it shifts the focus from learning to winning and has been associated with increased anxiety [58,59]. A recent randomized crossover study warned against using competitive gamification in medical education, finding no evidence of benefits on students’ competence or internal motivation [60].

Our use of free, open-access platforms addressed barriers in resource-limited settings, where 50% of gamified training platforms for preclinical medical education required paid access [61]. This aligns with the United Nations Educational, Scientific and Cultural Organization “Education for All” initiative by promoting equitable educational resources worldwide [62]. Moreover, while most research on gamification as an educational tool in health professions has been conducted in the United States or Canada [25], by conducting this study in the Middle East—an underrepresented region in gamification research—we contributed valuable data to global medical education literature and demonstrated the approach’s feasibility beyond North American contexts.

Further research is essential to build on our findings and to fully understand the potential of gamification in pharmacology education. Investigating the broader impact of open-access, web-based gamification will help validate and generalize these results, paving the way for more engaging and effective learning experiences for medical students. In future research, we would like to explore additional parameters such as academic achievement, long-term memory retention, and practical applicability. As a next step, we also propose developing a free mobile app. This tool has significant potential for learning due to its easy accessibility and affordability, especially because mobile apps are rarely used in pharmacology education [63].

Our study has several limitations. As a single-site, quasi-experimental study with a modest sample size, its generalizability may be limited. As students self-selected as gamers, it is possible that those who were more academically motivated chose the games as an additional study tool. There were also substantially more gamer instances than controls. To mitigate potential bias from unequal group sizes, we analyzed learning sessions rather than unique students, treating each week’s participation as an independent observation reflecting exposure to the intervention. We used nonparametric statistics (Mann-Whitney U test), which do not assume equal variances or normally distributed data, thereby making our comparisons robust to group-size differences. Future studies should use randomized allocation or stratified sampling to ensure balanced groups.

In conclusion, integrating simple, low-cost web-based games into a pharmacology curriculum can enhance knowledge retention and learner engagement. As a complementary, optional strategy, gamification offers a feasible strategy to enrich traditional didactics and meets the needs of diverse medical student populations.

## Supplementary material

10.2196/73666 Multimedia Appendix 1 Screenshots of the open-access digital pharmacology games developed for the study: *Cross DRUGs* (crossword puzzle), *Find the DRUG* (word search), and *DRUGs Escape Room* (interactive Google Form–based challenge).

10.2196/73666 Multimedia Appendix 2 Cronbach α tool for the level of satisfaction with gamification as a learning tool.

10.2196/73666 Multimedia Appendix 3 Demographics of the students who participated (gamers) and did not participate (control) in the games.

## Abbreviations

CONSORT-EHEALTH: Consolidated Standards of Reporting Trials of Electronic and Mobile Health Applications and Online Telehealth
UNESCO: United Nations Educational, Scientific and Cultural Organization
