# Supplementary material for: Open-Access Web-Based Gamification in Pharmacology Education for Medical Students: Quasi-Experimental Study
Source: JMIR Med Educ. 2025 Dec 5;11:e73666. doi: 10.2196/73666 (PMC12680091; doi:10.2196/73666)
Supplement: Multimedia Appendix 1 [file mededu-v11-e73666-s001.pdf]

Cross Drugs!

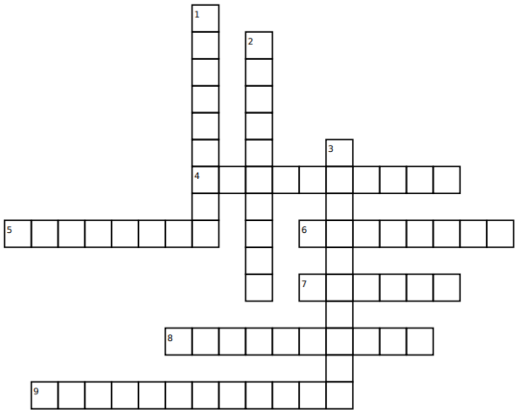

Down:
1. A drug with broadest anti-seizure spectrum of action
2. A drug with an atropine like effect
3. A drug that blocks ryanodine receptor

Across:
4. A drug with similar structure to thyroxine
5. A drug that causes vivid dreams
6. A drug used to treat bradycardia
7. A local anesthetic blocks this ionic channel
8. A drug that is a GABA antagonist
9. A drug that inhibits cholinesterase

Find the DRUG!

A	L	S	E	L	R	G	G	I	Q	K	M	N	E
M	U	Q	G	O	M	O	X	M	V	S	C	S	M
I	Z	U	C	R	T	E	Q	I	J	A	V	B	K
N	P	Q	L	A	D	I	X	P	B	L	P	T	N
O	P	K	O	T	A	C	W	R	S	M	R	C	V
S	E	G	N	A	M	G	K	A	I	E	A	I	U
T	D	B	I	D	G	C	C	M	M	T	M	M	U
E	J	T	D	I	U	F	X	I	J	E	I	E	W
R	H	L	I	N	F	M	K	N	P	R	P	T	F
O	B	D	N	E	B	L	E	E	B	O	E	I	L
I	M	F	E	F	P	C	I	O	M	L	X	D	N
D	B	U	S	P	I	R	O	N	E	T	O	I	Z
M	D	A	T	E	N	O	L	O	L	B	L	N	K
R	G	U	X	L	B	P	M	W	H	S	E	E	U

- A drug that acts on alpha 2 receptor
- A drug that is a sedating tricyclic antidepressant
- A drug that modified cyclodextrin can antagonize
- A drug used to treat seasonal allergy without causing sedation
- A drug that is water soluble (*hint: beta blocker*)
- A drug that is a long acting beta-2 agonist
- A drug that activates dopamine receptors
- A drug that is an agonist at 5-HT1A receptors
- A drug with anti-androgenic side effects

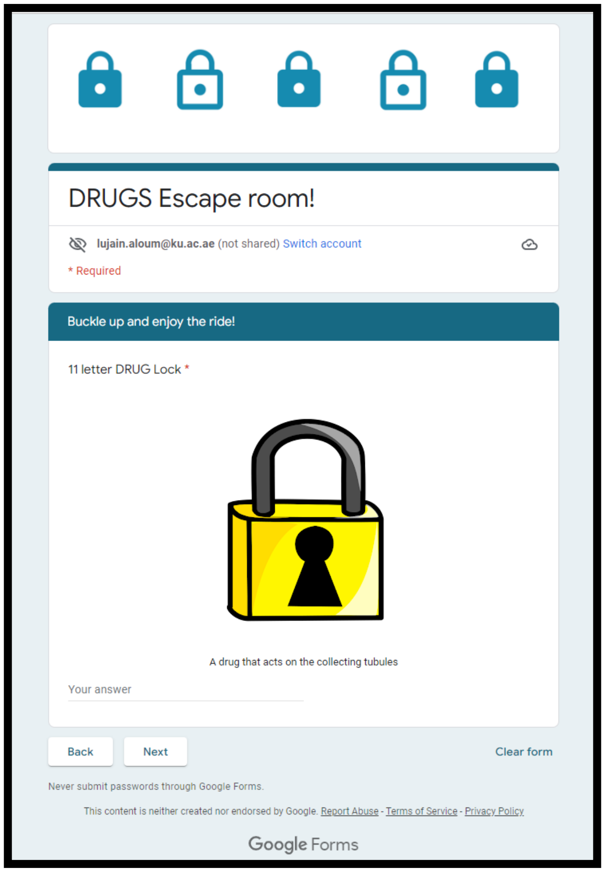

DRUGS Escape room!

lujain.aloum@ku.ac.ae (not shared) Switch account

* Required

Buckle up and enjoy the ride!

11 letter DRUG Lock *

A drug that acts on the collecting tubules

Your answer

Back Next Clear form

Never submit passwords through Google Forms.

This content is neither created nor endorsed by Google. Report Abuse - Terms of Service - Privacy Policy

Google Forms
