# Supplementary material for: Open-Access Web-Based Gamification in Pharmacology Education for Medical Students: Quasi-Experimental Study
Source: JMIR Med Educ. 2025 Dec 5;11:e73666. doi: 10.2196/73666 (PMC12680091; doi:10.2196/73666)
Supplement: Multimedia Appendix 2 [file mededu-v11-e73666-s002.pdf]

Item Description	Cronbach's Alpha if Item Deleted	Cronbach's Alpha
Effectiveness	0.79	0.88
Joyfulness	0.85	
Learning gain	0.86	
