# Supplementary material for: Open-Access Web-Based Gamification in Pharmacology Education for Medical Students: Quasi-Experimental Study
Source: JMIR Med Educ. 2025 Dec 5;11:e73666. doi: 10.2196/73666 (PMC12680091; doi:10.2196/73666)
Supplement: Multimedia Appendix 3 [file mededu-v11-e73666-s003.pdf]

	Group	Gender		Age (average in years)	Educational background		
		Male	Female		Engineering	Pharmacy/ Pharmacology	Others
**Game 1**							
	Control	4	5	23.9	3	0	6
	Gamers	11	14	23.8	10	0	15
**Game 2**							
	Control	2	3	24.4	1	0	4
	Gamers	6	12	23.7	9	0	9
**Game 3**							
	Control	3	3	23.5	0	0	6
	Gamers	6	10	24.4	8	0	8
**Total**							
	Control	9	11	23.9	4	0	16
	Gamers	23	36	24.0	27	0	32
